# Neurocognitive Performance in Adults Treated With Radiation for a Primary Brain Tumor

**DOI:** 10.1016/j.adro.2022.101028

**Published:** 2022-07-16

**Authors:** Derek S. Tsang, Mohammad M. Khandwala, Zhihui Amy Liu, Nadine Richard, Gerald Shen, Angela Sekely, Lori J. Bernstein, Rebecca Simpson, Warren Mason, Caroline Chung, Fabio Ynoe de Moraes, Louise Murray, David Shultz, Normand Laperriere, Barbara-Ann Millar, Kim Edelstein

**Affiliations:** a Radiation Medicine Program; b Department of Biostatistics; c Supportive Care, Princess Margaret Cancer Centre, University Health Network, Toronto, Canada; d Graduate Department of Psychological Clinical Science, University of Toronto, Toronto, Canada; e Division of Medical Oncology & Hematology, Princess Margaret Cancer Centre, University Health Network, Toronto, Canada; f Division of Radiation Oncology, MD Anderson Cancer Centre, Houston, Texas; g Department of Oncology, Queen's University, Kingston, Canada; h Radiotherapy Research Group, University of Leeds, Leeds, United Kingdom

## Abstract

**Purpose:**

The contributory effects of radiation dose to different brain regions on neurocognitive performance after radiation therapy (RT) for primary brain tumors is not well known.

**Methods and Materials:**

In this retrospective cohort study, 30 patients with brain tumors treated with photon RT were identified, and radiation dosimetric parameters across brain regions were calculated. All patients had longitudinal neurocognitive evaluations at baseline and after treatment. Generalized estimating equations were used to model each neurocognitive endpoint over time in a multivariable analysis, while adjusted for multiple comparisons of brain regions.

**Results:**

Median follow-up from RT to last assessment was 4.1 years. Fewer years of formal education and older age at the time of RT were associated with lower scores in language, verbal memory, and working memory, after adjustment for baseline scores in multivariable analyses. Higher radiation dose to specific brain regions was not associated with declines in any of the evaluated cognitive domains. On average, there was no clinically significant decline (magnitude of z score change >1) between first and last neurocognitive evaluation. Across each individual cognitive domain, fewer than 15% of patients were impaired at most recent follow-up.

**Conclusions:**

In this small study of 30 patients treated with RT for a primary brain tumor, brain region dosimetry was not associated with decline in cognitive performance. Older age at time of RT and fewer years of formal education were associated with declines in cognitive performance, suggesting that effects of nondosimetric factors on cognitive performance should be considered alongside treatment factors and dosimetry in neuro-oncology research.

## Introduction

Many patients with primary brain tumors have good long-term outcomes and survival after radiation therapy (RT), particularly individuals with curable histologies such as meningiomas,^1^ medulloblastoma,^2^ or some low-grade gliomas.^3^ As patients transition from active oncology follow-up to long-term survivorship care, potential treatment-related late effects take on increasing importance over time. There is increasing recognition of the neurocognitive consequences of therapy on adults after brain tumor treatment, and awareness of the contributions of different clinical and treatment factors is essential to develop ways to mitigate cognitive deficits.^4^^,^^5^

Tumor factors such as size or location,^6^^,^^7^ surgical resection,^8^ and RT^9^ and patient factors such as age^10^ are known to contribute to the risk of neurocognitive change after therapy. Because this change is multifactorial in etiology, it can be challenging to determine the individual contributions of each therapeutic exposure on neurocognition. Although research suggests that the developing and aging brains are particularly vulnerable to the effects of RT,^11^ much less is known about these effects in adults in midlife. Discrepancies in study design, timing of assessments, absence of pretreatment baseline assessments, tumor progression, and unidentified factors that contribute to neurocognitive change also make it challenging to understand individual risk for neurocognitive decline after RT. Moreover, the role of RT exposure to brain regions on higher order cognitive functions in adults – beyond the hippocampus and memory – is not well known in the setting of primary brain tumors.

The goal of this study was to calculate 3-dimensional radiation dosimetry to specific brain regions important for neurocognition and to evaluate dosimetry, clinical factors, and their associations with change in neurocognitive functions in the years after RT. To do this, we created models of neurocognitive change over time among adults treated with radiation for primary brain tumors and evaluated dosimetric associations with neurocognitive performance.

## Methods and Materials

This was a retrospective cohort study of patients treated at Princess Margaret Cancer Centre, University Health Network in Toronto, Canada. Patients were eligible for inclusion if they were age 16 years or older at time of RT, had histologic or radiologic evidence of a primary brain tumor (ie, not brain metastasis), were treated with fractionated external beam radiation with accessible dosimetry (ie, after 2004), and underwent a clinical neuropsychological assessment to establish a new baseline within 1 year before or after RT, and had at least 1 subsequent follow-up assessment. Because the aim of our study was to examine changes in cognition associated with the late effects of RT, patients who had tumor progression before undergoing a follow-up assessment were excluded. In addition, cognitive evaluations acquired after tumor recurrence post-RT were excluded. Eligible patients were identified from an adult neuropsychology referral database and eligibility was confirmed with details from clinical and RT charts. Patients were referred for baseline assessments by the treating radiation or neuro-oncologist at their discretion or by patient request; follow-up assessments were routinely offered to patients 1 to 2 years post-RT without evidence of tumor recurrence. Repeat evaluations were also conducted in response to physician or self-referral. This study was reviewed and approved by the University Health Network research ethics board.

### Radiation treatment and dosimetry evaluation

All patients were treated with linear accelerator-based photon therapy at a single institution. Patients who received whole brain or craniospinal irradiation were treated with field-based techniques, while those treated with focal or boost RT received conformal RT, such as intensity modulated radiation therapy or volumetric modulated arc therapy. No patient received memantine. Brain regions were contoured in the treatment planning system (Pinnacle; Koninklijke Philips N.V., Amsterdam, The Netherlands) with computed tomography/magnetic resonance fusion and overlaid with the clinical RT plan and dose in 3 dimensions. Volumes were segmented and checked by 1 observer and verified by a second observer. Example brain region contours on magnetic resonance brain imaging are shown in Fig. E1. Corpus callosum and hypothalamic-pituitary axis contours were drawn using publicly available atlases.^12^^,^^13^

### Neurocognitive assessments

Patients referred for clinical neuropsychological assessment by a member of their health care team completed standardized neuropsychological tests and validated measures of anxiety and depression administered by qualified staff. At follow-up evaluation, patients completed the same measures as baseline (see Table E1 for list of measures). Test scores were converted to age-corrected scaled scores according to published criteria and transformed to z scores (mean, 0; standard deviation [SD], 1). Tests that measure the same cognitive domain were averaged to create composite scores: verbal and visuospatial skills, processing speed, attention, working memory, executive function, motor dexterity, verbal memory, and visual memory (Table E1). Mean change scores (Δz) were calculated by subtracting z scores from first to last evaluation for each test and averaged for each domain. We defined clinically meaningful change between first and last assessment across each domain as a z score difference greater than 1 in magnitude (ie, 1 SD)^14^; these changes were further categorized by magnitude (≥1 and <1.5; ≥1.5 and <2; ≥2). Patients were classified as having cognitive impairment at most recent evaluation if they had at least 1 test with a z score that was at least 2 SD below the normative mean (ie, z ≤ -2) or 2 or more tests that were at least 1.5 SD below the mean (ie, z ≤ -1.5), consistent with International Cancer and Cognition Task Force criteria.^15^

### Analysis

Generalized estimating equations (GEE) were used to model each neurocognitive endpoint over time in a multivariable analysis, to account for varying timepoints of evaluation with respect to treatment exposures. This model was able to account for patient-specific changes over time as well as baseline function at first evaluation. Dosimetric variables chosen a priori included mean and dose to 50% (D50) of the brain and brain minus the gross tumor volume, mean, D50 and D40 to the left/right hippocampi, and mean doses to the brain stem and cerebellum, left/right ventral frontal cortex, left/right dorsofrontal cortex, subcortical brain, left/right parieto-occipital cortex, and left/right temporal lobe (Fig. E1). Nondosimetric variables chosen a priori included: baseline neurocognitive performance (of the modelled domain), patient age at time of RT, time since RT, and years of formal education. Exploratory analyses were also performed with respect to corpus callosum and hypothalamic-pituitary axis dosimetry. *P* values for dosimetric variables were adjusted for multiple comparisons using the false discovery rate method denoted as the q-value, to account for the number of brain regions tested during GEE modeling. To further explore neurocognitive impairment after treatment, we completed a cross-sectional analysis of the most recent neurocognitive evaluation across all patients. Cognitive impairment was defined as a binary endpoint as previously mentioned, as per International Cancer and Cognition Task Force criteria. A threshold of <0.05 was used for statistical significance. Statistical analyses were performed using R version 3.5.2 (R Foundation for Statistical Computing, Vienna, Austria).

## Results

Thirty patients met inclusion criteria. Baseline demographic, clinical, and tumor characteristics are listed in Table 1. Patients were treated with radiation between 2004 and 2013. Dosimetric parameters for all evaluated structures, stratified by field of RT, are reported in Table E2.

**Table 1:** Characteristics of patient cohort

Characteristics	n = 30
Female sex (%)	14 (47)
Age at diagnosis, years, median (range)	39 (17-63)
Age at RT, years, median (range)	42 (17-71)
Right-handed (%)	28 (93)
Formal education at first evaluation, total years, median (range)	15 (3-23)
Marital status (%)	
Married	16 (53)
Living with parent(s)	8 (27)
Single	4 (13)
Separated or divorced	2 (7)
Depression, BDI-II, mean ± SD (range)	14.7 ± 10.2 (3-48)
Moderate depression (BDI-II ≥20)* (%)	16 (57)
Anxiety, STAI-S, mean ± SD (range)	50.7 ± 10.6 (36-80)
Experiencing anxiety (STAI-S ≥65)† (%)	3 (11)
Diagnosis (%)	
Meningioma	11 (37)
Medulloblastoma‡	6 (20)
High-grade glioma	2 (7)
Low-grade glioma	2 (7)
Primary CNS lymphoma	2 (7)
Germinoma	2 (7)
Craniopharygioma	2 (7)
Other§	3 (10)
Tumor volume at time of RT, cc, median (range)	23.2 (2.1-90.6)
Number of surgeries before first neurocognitive evaluation	
None	3
1	18
2	5
≥3	4
Systemic therapy (%)	10 (33)
Temozolomide exposure	5 (50)
Cisplatin exposure	3 (30)
Methotrexate exposure║	2 (20)
RT	30 (100)
Focal (%)	19 (63)
Whole brain¶ (%)	2 (7)
Whole brain¶ with boost (%)	9 (30)
Whole brain dose, Gy, median (range)	36 (25-54)
Total dose, Gy, median (range)	54 (40-70)
Number of fractions, median (range)	30 (20-40)
30 fractions (%)	12 (40)
25 fractions (%)	7 (23)
33 fractions (%)	6 (20)
Antiepileptic drugs (%)	
At first evaluation only#	3 (10)
At first and follow-up evaluations⁎⁎	4 (13)

*Abbreviations:* BDI = Beck depression inventory; CNS = central nervous system; RT = radiation therapy; SD = standard deviation; STAI-S = state-trait anxiety inventory, S-anxiety subscale.

⁎ Two patients had missing data.

† Three patients had missing data.

‡ Includes 1 pineoblastoma.

§ One case each of chondrosarcoma, brain sarcoma, hemangiopericytoma.

║ Methotrexate exposure preceded first neurocognitive evaluation in both patients.

¶ Includes craniospinal irradiation.

# Phenytoin (n = 3).

⁎⁎ Carbamazepine (n = 2), lacosamide and levetiracetam (n = 1), phenytoin (n = 1).

Patients completed their baseline evaluations between 8 weeks before RT and 42 weeks after RT, with a median time between the first evaluation and RT of 2.6 weeks. The median follow-up time from initial tumor diagnosis to last neurocognitive assessment was 5.6 years (range, 0.9-22.8). The median time from RT to last assessment was 4.1 years (0.5-13.7). The median time from first to last neurocognitive assessment was 3.9 years (0.5-13.8). A total of 30, 8, and 4 patients had 2, 3, or 4 neurocognitive evaluations, respectively. A plot of the timing of neurocognitive assessments relative to RT is provided in Fig. E2. Ten patients (36%) experienced tumor recurrence after their most recent neurocognitive evaluation (median time from last assessment to recurrence was 20 months [range, 3-64]).

**Figure 1:**
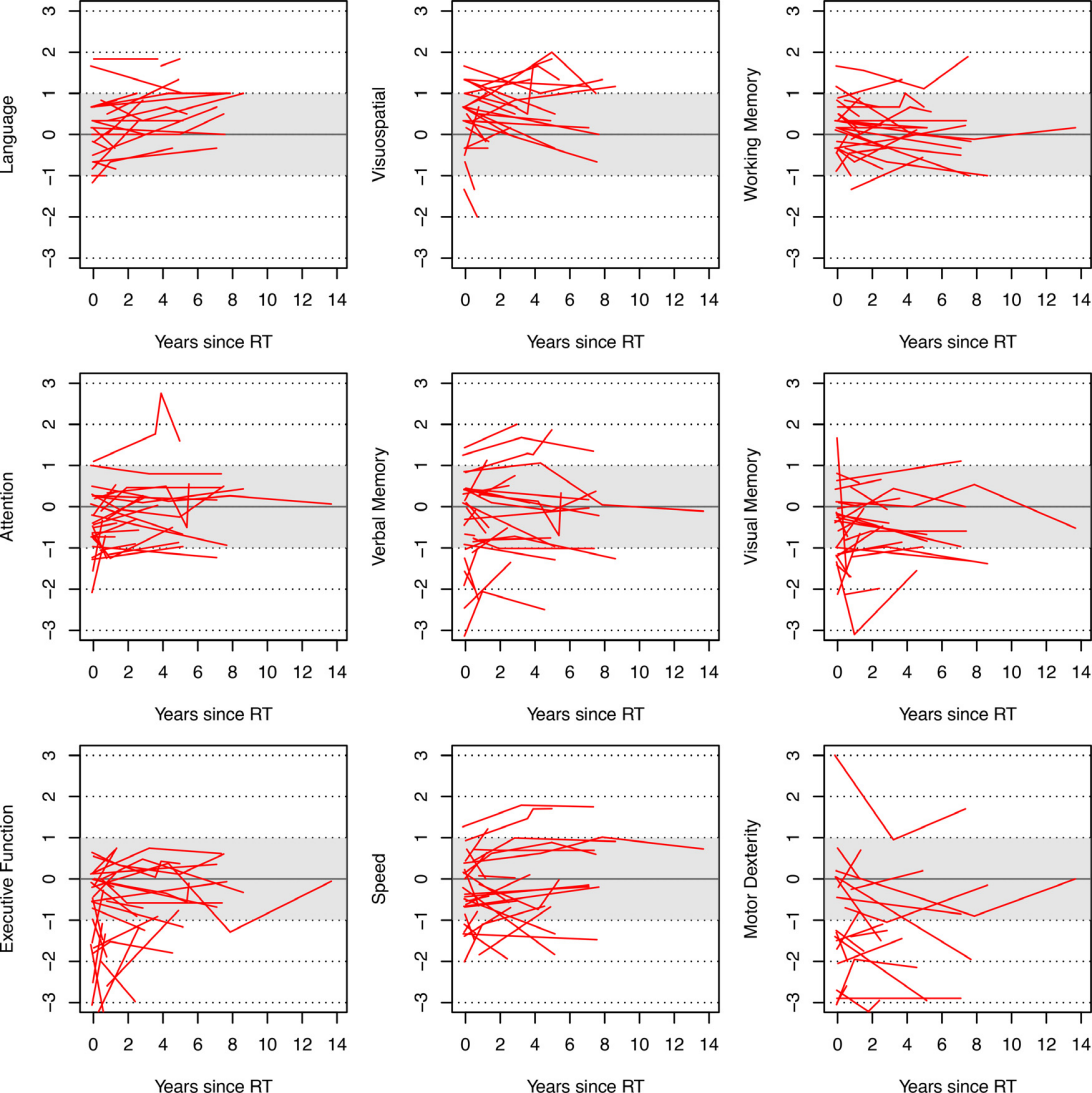
Plots of neurocognitive domain z scores over time since radiation therapy. Higher scores indicate better performance. A score of zero is equal to the population mean, with a population standard deviation of ±1 (gray shaded region). These plots are truncated beyond -3 and +3 on the vertical axis; for the full range of values displayed, please see Fig. E3.

Neurocognitive performance at baseline and follow-up is shown in Fig. 1. Most scores were within the average range except for executive functions and motor dexterity but varied considerably across domains, individuals, and over time. In Table 2, the GEE model is presented, which incorporates baseline neurocognitive function, time since RT, patient age at RT, years of formal education, and RT dose to brain regions. A key finding was that increasing RT doses to any evaluated region or the whole brain were not associated with decline in any cognitive domain after adjustment for the other considered factors (listed in Table 2). For the cognitive domain of speed only, increased dose to the hypothalamic-pituitary axis was associated with improved speed. No other cognition-dose associations were found.

**Table 2:** Generalized estimating equation models of neurocognitive function across different cognitive domains.

Cognitive domain	Evaluable	Baseline z score		Baseline function (z score)		Time since RT (y)		Age at RT (y)		Formal education (y)		Dose to structure (per Gy)			
	n	Mean	SD	ß	*P*	ß	*P*	ß	*P*	ß	*P*	Structure	Metric	ß	q
Attention	29	–0.4	0.7	None significant											
Executive function	29	–0.8	1.1	–0.128	.74	–0.122	.22	–0.068	**.050**	0.007	.91	Left parieto-occipital	Mean	–0.005	0.77
Verbal abilities (language)	30	0.2	0.9	–0.348	**< .001**	0.054	**.027**	–0.014	**.021**	0.050	**.043**	Left temporal	Mean	–0.009	0.51
Motor dexterity	25	–1.2	1.6	–0.367	**< .001**	0.003	.95	–0.042	**.002**	0.070	**.042**	Infratentorial brain	Mean	–0.015	0.18
Speed (model 1)	29	–0.5	1.3	–0.118	.16	0.031	.26	–0.017	**.022**	0.043	.15	Left hippocampus	D40	0.011	0.18
Speed (model 2)	30	–0.5	1.3	–0.130	.098	0.012	.62	–0.015	**.047**	0.039	.16	Hypothalamic-pituitary axis	Mean	0.013	**0.0039**
Verbal memory	29	–0.3	1.1	–0.133	.10	–0.075	**.012**	–0.010	.31	0.041	**.043**	Left dorsal frontal	Mean	0.003	0.92
Visual memory (model 1)	29	–0.6	0.9	–0.590	**.029**	0.030	.50	–0.017	.10	–0.020	.70	Subcortical	Mean	–0.017	0.54
Visual memory (model 2)	29	–0.6	0.9	–0.496	**.044**	0.037	.43	–0.008	.27	–0.021	.67	Left hippocampus	Mean	–0.010	0.54
Visuospatial skills	30	0.4	0.8	None significant											
Working memory	30	0.1	0.7	–0.205	.158	–0.056	**.005**	–0.002	.746	0.059	**.004**	Left ventral frontal	Mean	–0.010	0.52

*Abbreviations:* RT = radiation therapy; SD = standard deviation.

Negative coefficients (ß) denote worse performance associated with increasing magnitude of that variable. In this table for each cognitive domain, we included the structure that had the lowest q-value. There were no statistically significant associations found with any of the listed variables in the attention and visuospatial domains. Bolded values denote statistical significance (p or q < 0.05).

Age at the time of RT and years of formal education were associated with 4 cognitive domains each (language and motor dexterity with both; executive function and speed with age at RT; verbal memory and working memory with years of formal education; see Table 2 for details). Increasing age was associated with a *decline* in cognitive performance, while increasing years of formal education were associated with *better* cognitive performance (in the setting of postradiation follow-up). In the multivariable GEE model, the passage of time from treatment was associated with statistically significant declines in 2 cognitive domains (verbal memory and working memory), while language function improved with time. Finally, baseline function remained an important determinant of performance in language, motor dexterity, and visual memory. Individuals with higher baseline function experienced more severe declines. Results from the GEE model were largely unchanged after excluding 1 patient with delayed baseline cognitive evaluation at 42 weeks post-RT (Table E3). In an exploratory analysis of 19 patients who received focal RT alone, baseline function, age at RT, and years of formal education continue to be associated with multiple cognitive domains (Table E4). Higher D40 to the left hippocampus was associated with faster speed; no other associations with dosimetry were found.

**Figure 2:**
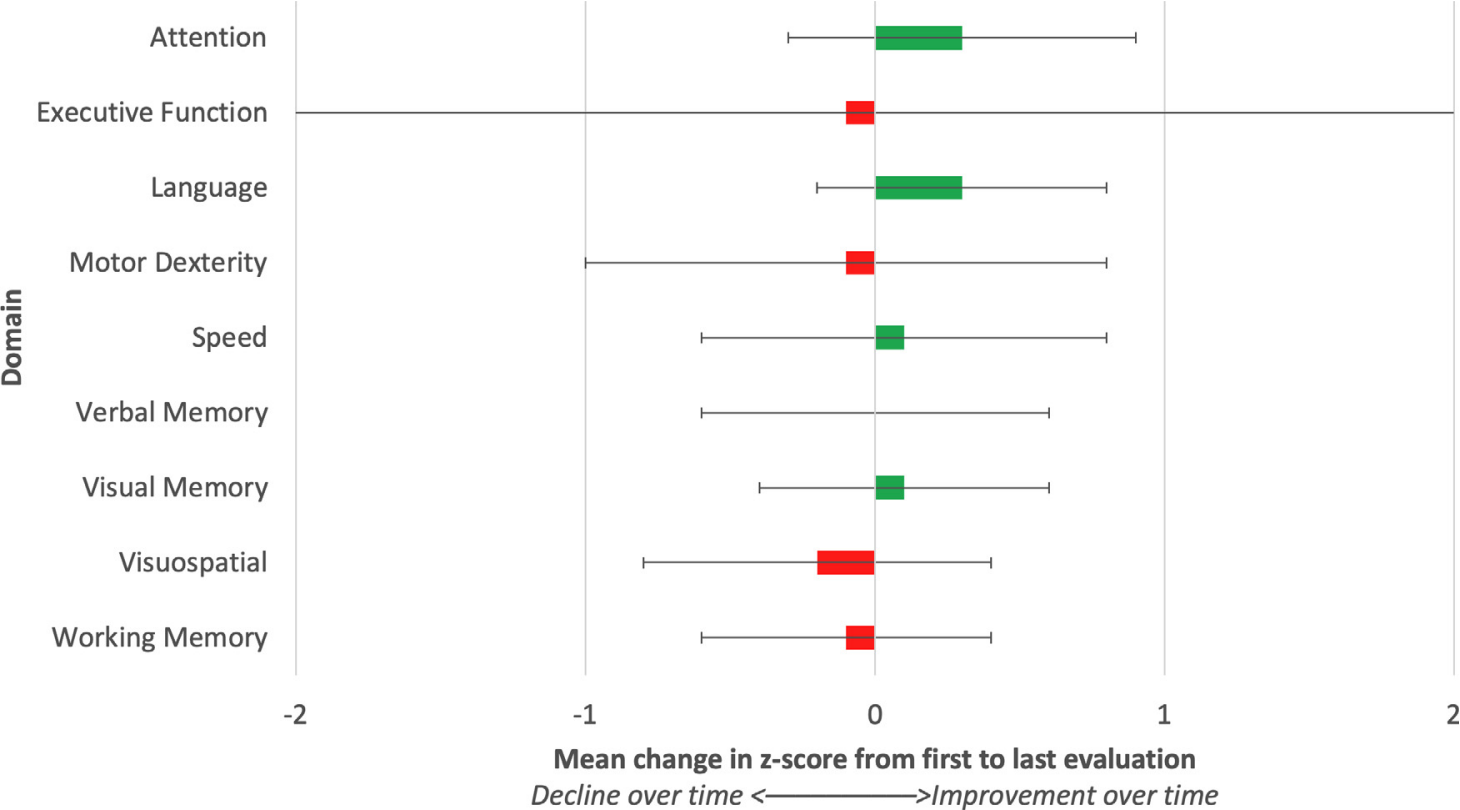
Comparison of mean change in z scores, across all patients, from first to last evaluation. Positive scores indicate improvement in domain score (ie, better cognition), while negative scores indicate a decline in cognition. The standard deviation is denoted by the error bars; 68.3% of values fall within the error bars. Because 1 patient had a large decrease in executive function at follow-up, standard deviation was large for this domain.

Patients generally retained cognitive function across most domains at their most recent neurocognitive evaluation (Fig. 1), though there was considerable variability across patients, particularly in executive function and motor dexterity. On average, there were no clinically significant changes in z scores across domains between first to last evaluation (Fig. 2), as no domain had an average z score change greater than 1.0. A few individual patients did experience z score declines across domains (Table E5); the domain with the greatest number of patients experiencing decline between first and last evaluation was in motor dexterity (n = 4 having a z score decline of greater than 1.0).

**Figure 3:**
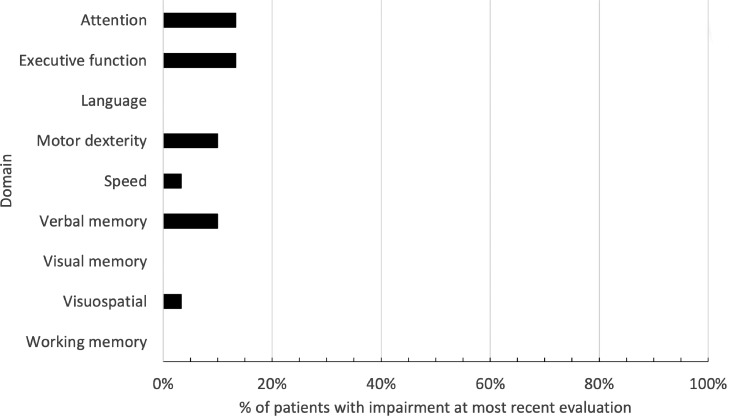
Proportion of patients demonstrating cognitive impairment at most recent evaluation, as per International Cancer and Cognition Task Force criteria.

Figure 3 plots the proportion of patients impaired in each domain at most recent follow-up. No patients experienced known impairment in language, visual memory, or working memory. Four patients were impaired in attention or executive function, 3 patients were impaired in verbal memory or motor dexterity, and 1 patient was impaired in visuospatial performance or speed.

## Discussion

In this study of 30 adults treated with RT for a primary brain tumor, longitudinal neurocognitive follow-up demonstrated the importance of patient demographic factors in determining cognitive function over time, including age at RT and years of formal education. Baseline performance was also associated with cognition at follow-up, with higher-performing individuals in selected domains experiencing greater declines in function post-RT. The proportion of patients showing neurocognitive impairment across domains was low, with no more than 15% of patients impaired at most recent follow-up in any domain. Few patients experienced decline in function. However, our median follow-up was only 4 years; cognitive changes may yet be seen with longer-term follow-up.

The consideration of nondosimetric factors on neurocognitive performance is important in the analysis of the side effects of cancer treatment. In a study by Rijnen et al^16^ with greater than 200 patients with meningioma, stronger cognitive performance postsurgery was associated with younger age and higher educational level, similar to the present study. Neurotoxicity is broadly recognized as more severe in elderly patients (over age 60) treated for primary central nervous system lymphoma, which is likely an effect of the lymphoma on brain function, as well as systemic therapy (methotrexate) and whole brain RT.^17^

RT to the brain is known to be associated with cognitive changes over time. However, this is likely a complex, multifactorial problem; other factors, such as the ability of RT to control tumor progression and prevent tumor-related cognitive decline, likely have a contributory effect. In this cohort of patients, we detected an association between higher hypothalamic-pituitary axis dose and greater speed in 1 of the GEE models. In addition, in an unplanned subgroup analysis of patients treated with focal RT alone, higher left hippocampus D40 was associated with greater speed. These associations are unusual^18^^,^^19^; we speculate this could potentially represent the benefits of tumor control, but these are findings in need of validation in independent, ideally prospective cohorts.

In addition, prolonged follow-up beyond 4 years may be required to observe detectable changes in cognitive function. In the European Organisation for Research and Treatment of Cancer 22033-26033 study, patients who received RT had slower recovery of memory posttreatment, but there were no differences in memory performance between those who did or did not receive RT at 12-months posttreatment.^20^ Two studies demonstrate the importance of long-term follow-up of brain tumor survivors. In the first study by Klein et al^21^ of 195 adults with supratentorial low-grade glioma (6 years mean follow-up), poor memory was only observed in patients who had daily fraction sizes >2 Gy per day. A follow-up study of the 65 patients who remained stable with a mean follow-up duration 12 years subsequently revealed patients were at risk of attention deficits, executive dysfunction, and slow processing speed, regardless of RT fraction size.^22^ The implication is that most adult patients do not manifest neurocognitive change due to RT until many years after treatment, consistent with findings in long-term pediatric brain tumor survivors.^23^ A limitation of this pair of studies was that baseline cognitive evaluations were not available, whereas this data was available in the present work.

Some individual patients in our study did experience declines in cognitive performance over time. Zureick et al^24^ found that higher hippocampal volume receiving 20 Gy was associated with declines in verbal and visual memory scores in children. Beyond visual memory, other studies have found that hippocampal dosimetry is important for cognitive functioning and memory. In a cross-sectional study of adults with brain tumors by Haldbo-Classen et al,^9^ higher left hippocampus RT dose was associated with lower scores on the Hopkins verbal learning test-revised. Similarly, work from Acharya et al^25^ and Tsang et al^26^ demonstrated associations between left hippocampus dosimetry with memory and verbal comprehension in children, respectively. In a large cohort of 124 children with medulloblastoma, mean hippocampus dose (right and left) was associated with declines in associative memory, while mean corpus callosum and frontal white matter doses were associated with declines in processing speed.^27^ Gondi et al^28^ found that left hippocampus dose was associated with verbal memory in adults with low-grade glioma, though their model was not validated in a subsequent study.^9^

Extensive work has been done in the realm of neurocognition after whole brain RT for brain metastases. Ma et al^29^ found a relationship between dose to 50% of the hippocampus and delayed recall. In a landmark randomized study, hippocampal avoidance during whole brain RT was associated with less cognitive failure compared with standard whole brain RT; both arms received memantine.^30^ Therefore, the hippocampus may be a region to be avoided during RT, if dosimetrically feasible. We did not find an association with hippocampal dosimetry across all patients in our small study, but our findings do confirm the clinical relevance of patient factors including age, baseline cognitive function, and years of education. Our study may also have been underpowered to detect weaker associations with hippocampal dosimetry and was unable to account for differences in tumor location; such associations may yet be detected with longer follow-up in larger patient samples.

Our finding that higher baseline function was associated with more decline in cognition after RT suggests that these patients have “more to lose” after treatment. In other words, those with stronger performance at baseline end up with steeper declines in cognition, whereas patients who are already mildly impaired at baseline experience less change. Other studies in the literature have remarked that those with impairment at baseline have greatest opportunity for improvement after tumor-directed therapy, which could be another explanation for our findings.^16^ In addition, patients who score extremely well at initial evaluation may be experiencing “regression to the mean,” leading to subsequent scores that are closer to population averages.^31^ The association of high baseline score and greater decline in performance is hypothesis-generating and requires validation in other data sets. If validated, then the role of interventions to preserve high baseline cognitive function after RT should be studied further.

There are limitations to our data. The total sample size was only 30 patients, limiting our ability to model the effect of a large number of factors on domain scores over time, including other variables such as anxiety and depression scores (which were not specified a priori in our analysis). Although we did not find strong associations with dosimetry to brain regions and cognitive performance, our study was underpowered to find weaker associations that may exist or may manifest after prolonged follow-up. However, our data do emphasize the importance of examining non-RT factors and cognitive change. In addition to age at RT and years of education, other non-RT treatment factors may influence cognitive outcomes, including surgery,^16^^,^^32^ surgical approach,^33^ direct tumor effects and tumor type,^34^ and systemic therapy.^17^^,^^35^ Unfortunately, our data were not able to evaluate all of these factors together due to a limited sample size. Some patients had their baseline assessment a few weeks after RT, though this was accounted for in the GEE model. Finally, in healthy people, improvements in cognitive performance occur due to practice effects, but also the likelihood of abnormally low scores increases with the number of tests administered.^15^^,^^36^ In future studies, including a cohort of healthy adults with similar age and education levels who do not have brain tumors as a control group may be helpful to examine reliable change over a similar time frame.^37^ Efforts to minimize potential bias in patients offered cognitive testing (ie, specific tumor types such as lower-grade tumors, younger patients, and those fluent in English) is also essential to ensure generalizable results.

Future work should include finding ways to determine who benefits the most from each specified treatment so that care is personalized. For example, patients with 1p19q-codeleted gliomas may be able to receive upfront systemic therapy, with deferred RT.^38^^,^^39^ There are 2 ongoing studies by NRG Oncology and the European Organisation for Research and Treatment of Cancer for patients with gross total resection of World Health Organization grade 2 meningioma, to evaluate the role of observation versus adjuvant RT (NCT03180268 and ISRCTN71502099). These studies incorporate longitudinal neurocognitive evaluations to better study changes after treatment. Other ways to mitigate cognitive decline posttreatment are needed. The role of cognitive rehabilitation has been studied in a randomized study, which demonstrated some positive effect on memory at 6-month follow-up.^40^ Similarly, cognitive rehabilitation through goal management training improved executive function in brain tumor survivors,^41^ though rehabilitation programs can be resource-intensive. None of the patients on this present study received memantine, a neuroprotectant that has been demonstrated to delay cognitive decline in patients treated with whole brain RT for brain metastases.^42^ The role of memantine has not been well studied in patients with primary brain tumors, however. Finally, preliminary work in pediatric brain tumor survivors has demonstrated promising results with metformin^43^ or physical exercise^44^ as neuroprotective, though studies in adults are needed.

## Conclusions

Among 30 adults treated with RT for a brain tumor, older age at the time of RT and fewer years of formal education were associated with declines in 4 cognitive domains each (age: executive function, language, motor dexterity, and speed; education: language, motor dexterity, verbal memory, and working memory). Dosimetry to brain regions was not associated with cognitive decline, though larger data sets and longer follow-up are needed to reveal potential associations. Patient baseline factors, such as age and prior education, are important to consider alongside RT dosimetry in neuro-oncology late effects research. Future research should focus on mitigating the effect of tumor-directed therapy upon cognition in brain tumor survivors.

## Appendix Supplementary materials

Image, application 1

Image, application 2

## Footnotes

Supplementary material associated with this article can be found in the online version at doi:10.1016/j.adro.2022.101028.
