# Supplementary material for: Neurocognitive Performance in Adults Treated With Radiation for a Primary Brain Tumor
Source: Adv Radiat Oncol. 2022 Jul 16;7(6):101028. doi: 10.1016/j.adro.2022.101028 (PMC9677214; doi:10.1016/j.adro.2022.101028)
Supplement: Supplementary file 1 [file mmc1.pptx]

## Slide 1
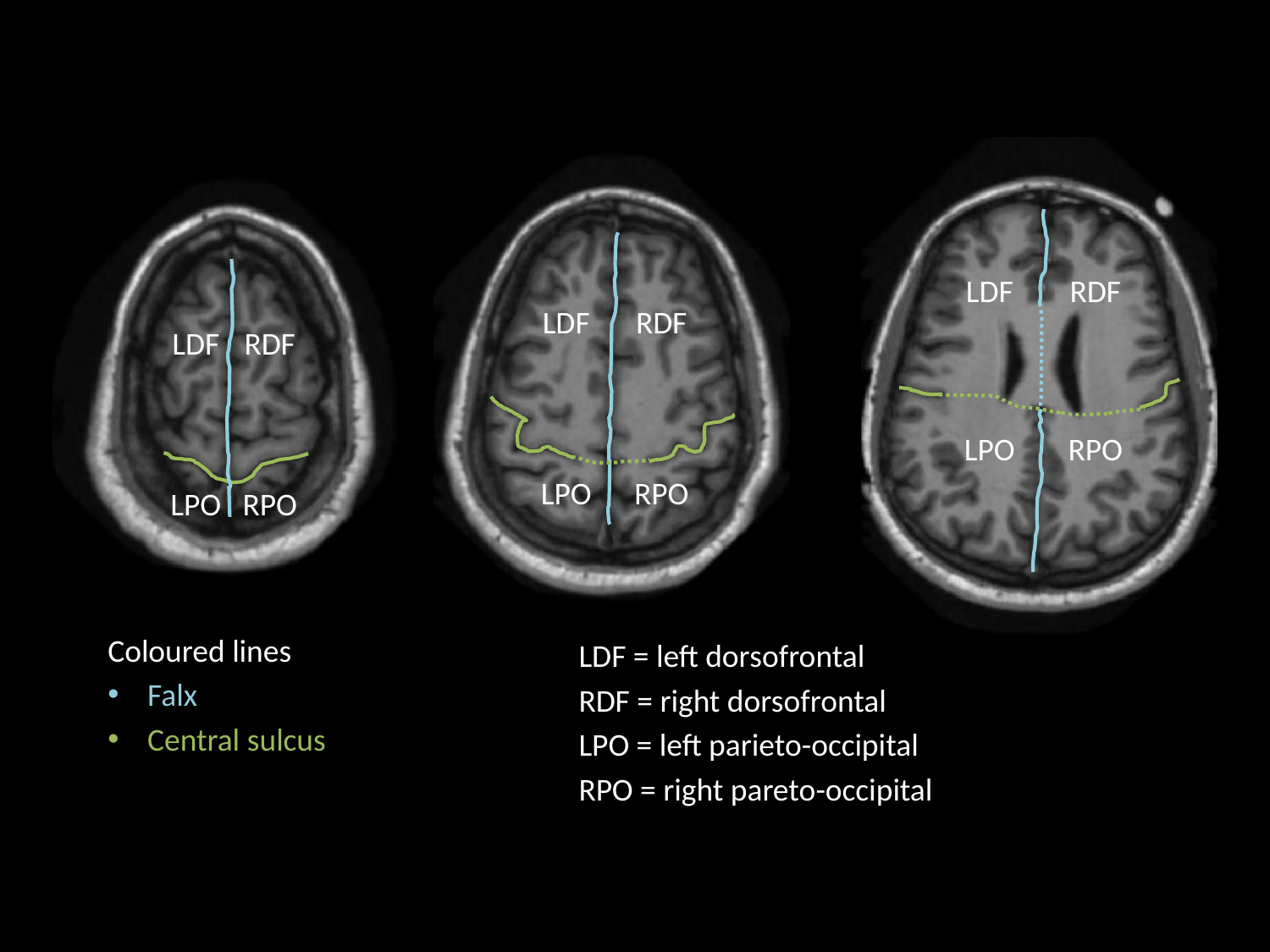

LDF
RDF
LDF
RDF
LDF
RDF
LPO
RPO
LPO
RPO
LPO
RPO
Coloured lines
Falx
Central sulcus
LDF = left dorsofrontal
RDF = right dorsofrontal
LPO = left parieto-occipital
RPO = right pareto-occipital

## Slide 2
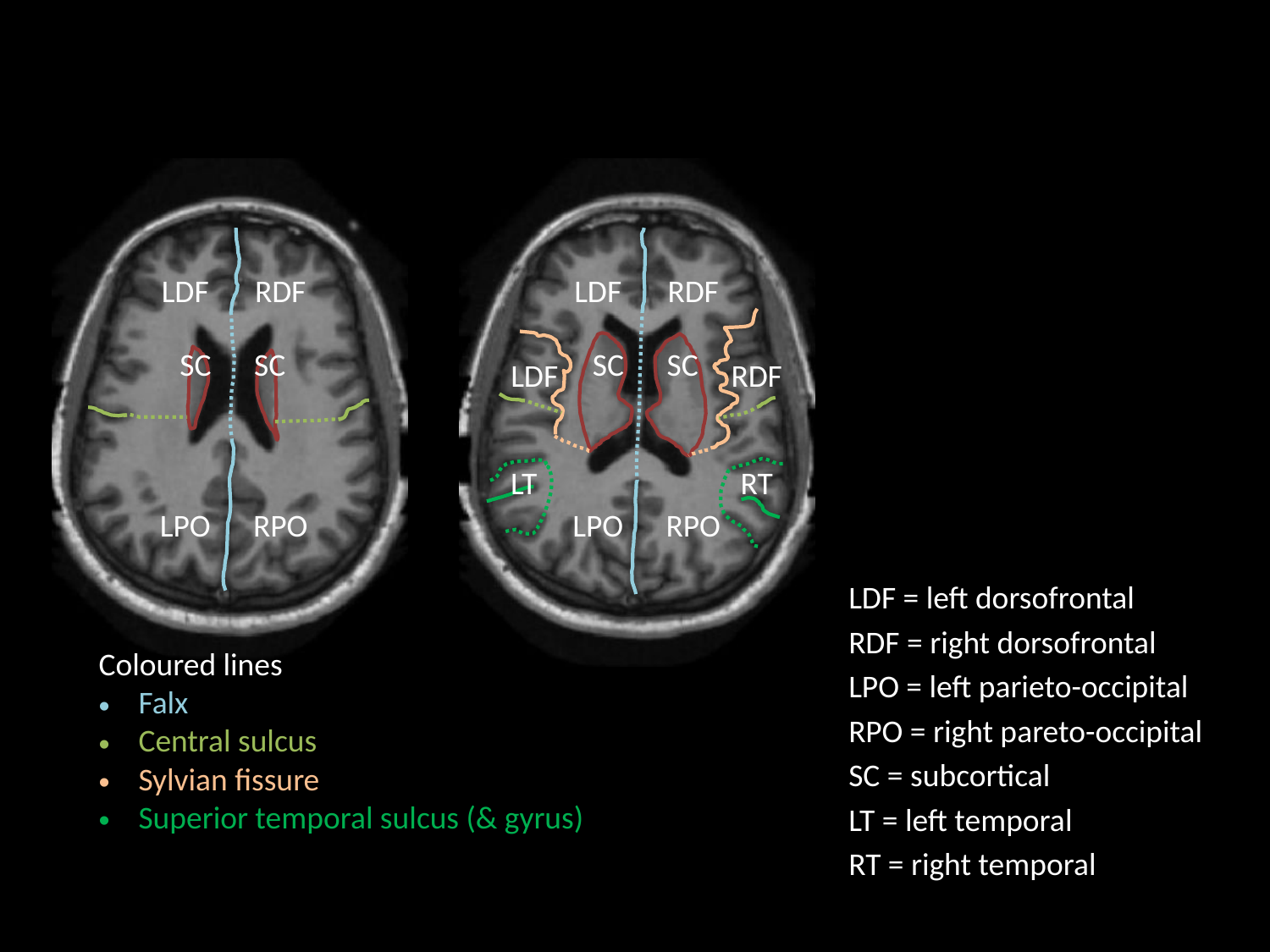

LDF
RDF
LDF
RDF
SC
SC
SC
SC
LDF
RDF
LT
RT
LPO
RPO
LPO
RPO
LDF = left dorsofrontal
RDF = right dorsofrontal
LPO = left parieto-occipital
RPO = right pareto-occipital
SC = subcortical
LT = left temporal
RT = right temporal
Coloured lines
Falx
Central sulcus
Sylvian fissure
Superior temporal sulcus (& gyrus)

## Slide 3
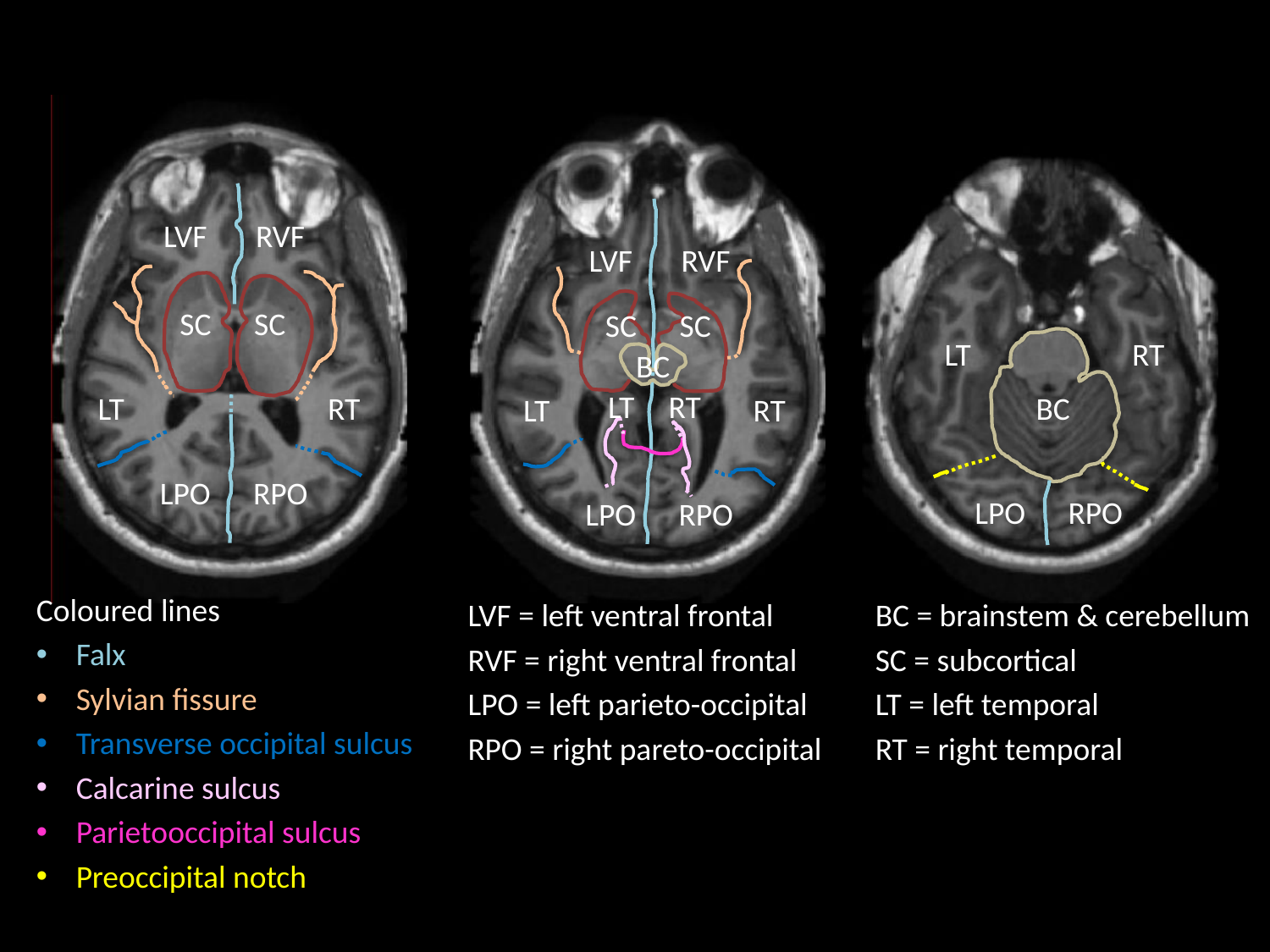

LVF
RVF
LVF
RVF
SC
SC
SC
SC
LT
RT
BC
LT
RT
LT
RT
BC
LT
RT
LPO
RPO
LPO
RPO
LPO
RPO
Coloured lines
Falx
Sylvian fissure
Transverse occipital sulcus
Calcarine sulcus
Parietooccipital sulcus
Preoccipital notch
LVF = left ventral frontal
RVF = right ventral frontal
LPO = left parieto-occipital
RPO = right pareto-occipital
BC = brainstem & cerebellum
SC = subcortical
LT = left temporal
RT = right temporal

## Slide 4
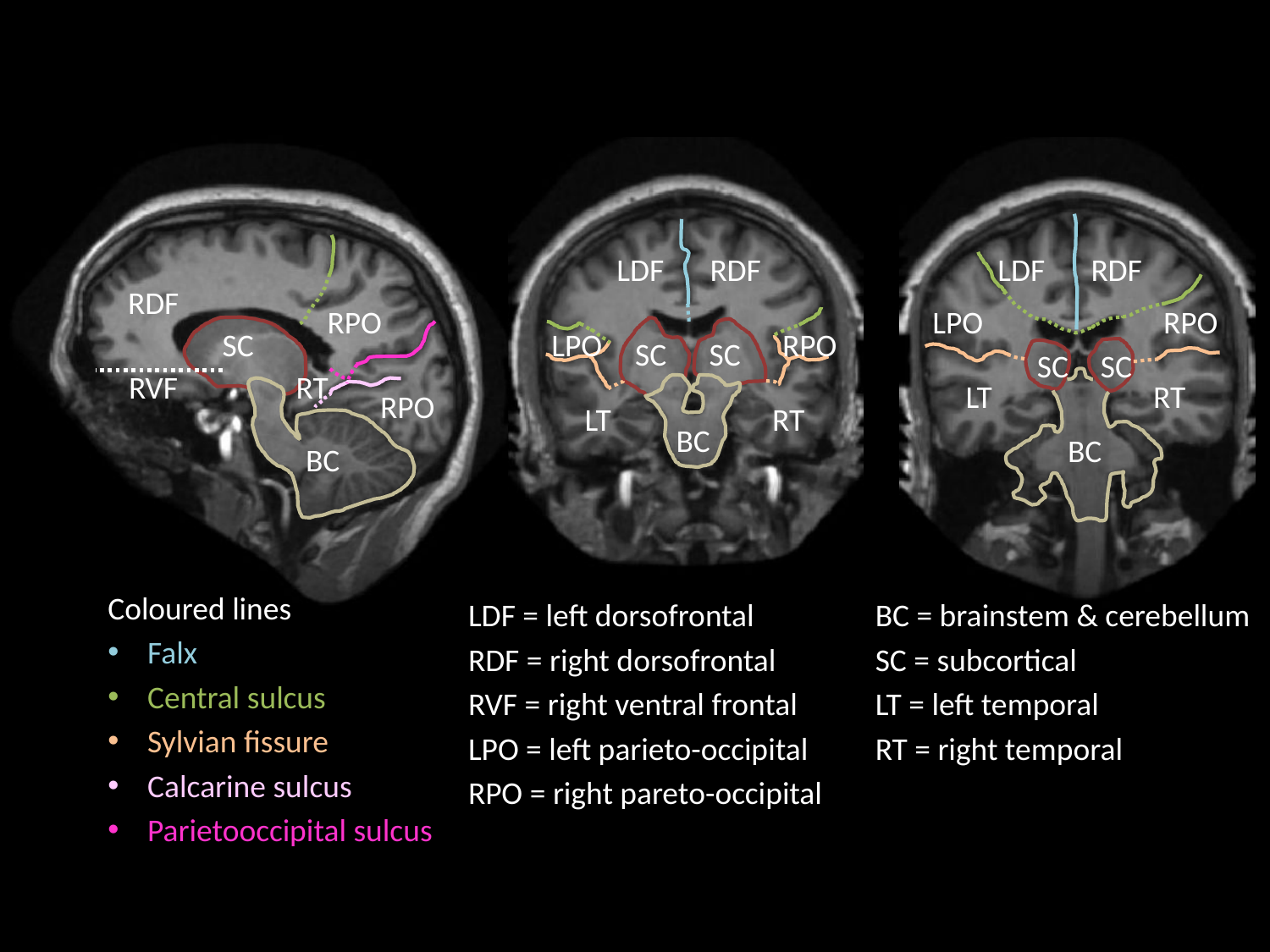

LDF
RDF
LDF
RDF
RDF
RPO
LPO
RPO
SC
LPO
RPO
SC
SC
SC
SC
RVF
RT
LT
RT
RPO
LT
LT
RT
BC
BC
BC
Coloured lines
Falx
Central sulcus
Sylvian fissure
Calcarine sulcus
Parietooccipital sulcus
LDF = left dorsofrontal
RDF = right dorsofrontal
RVF = right ventral frontal
LPO = left parieto-occipital
RPO = right pareto-occipital
BC = brainstem & cerebellum
SC = subcortical
LT = left temporal
RT = right temporal
