# Supplementary material for: Neurocognitive Performance in Adults Treated With Radiation for a Primary Brain Tumor
Source: Adv Radiat Oncol. 2022 Jul 16;7(6):101028. doi: 10.1016/j.adro.2022.101028 (PMC9677214; doi:10.1016/j.adro.2022.101028)

**Supplementary Table 1**. List of psychological testing measures used to evaluate neurocognitive domains

| **Neurocognitive Domain** | **Standardized Test** |
| --- | --- |
| Verbal Abilities/language (estimated verbal IQ) | Wechsler Abbreviated Scale of Intelligence^1^ (Vocabulary, Similarities) |
| Visuospatial Skills (estimated performance IQ) | Wechsler Abbreviated Scale of Intelligence^1^ (Block Design, Matrix Reasoning) |
| Attention | Test of Everyday Attention^2^ (Elevator search with distraction; Telephone search; Telephone search with distraction) |
|  | California Verbal Learning Test, 2^nd^ edition^3,4^ (List A trial 1; List B) |
| Working Memory | Wechsler Adult Intelligence Scale^3^ (Digit Span, Letter-Number Sequencing, Arithmetic) |
| Executive Functions | Verbal fluency (FAS)^5^ |
|  | Trailmaking Test, Part B^6^ |
|  | Conditional Associative Learning Test (Errors) |
|  | Wisconsin Card Sorting Test^7^ (Perseverative Responses, Errors) |
|  | Stroop (Interference) |
| Motor Dexterity | Grooved Pegboard |
| Processing Speed | Wechsler Adult Intelligence Scale^3^ (Digit Symbol Coding, Symbol Search) |
|  | Trailmaking Test, Part A |
| Verbal Memory | California Verbal Learning Test, 2^nd^ edition^3,4^ (Total Recall, Short Delay Free Recall, Long Delay Free Recall) |
|  | Wechsler Memory Scale, 3^rd^ edition (Logical Memory) |
| Visual Memory | Wechsler Memory Scale, 3^rd^ edition (Faces) |
|  | Doors and People (The Doors Test) |
|  | Rey-Osterreith Complex Figure (immediate, delayed recall) |
|  |  |
| **Self-reported Concerns** | **Standardized Questionnaire** |
| Depression | Beck Depression Inventory, 2^nd^ edition |
| Anxiety | Spielberger State/Trait Anxiety Inventory (STAI-S) |

^1^ Wechsler, D: The Wechsler Abbreviated Scale of Intelligence, San Antonio, TX, The Psychological Corporation, 1999

^2^ Robertson IH, Ward T, Ridgeway V, Nimmo-Smith I. Test of Everyday Attention. Suffolk, England: Thames Valley Test Company; 1994.

^3^ Wechsler D. Wechsler Adult Intelligence Scale-Fourth Edition. San Antonio, Texas: Pearson; 2008.

^4^ Delis DC, Kramer JH, Kaplan E, et al: California Verbal Learning Test - Adult Version (ed 2). San Antonio, TX, The Psychological Corporation, 2000

^5^ Tombaugh TN, Kozak J, Rees L. Normative data stratified by age and education for two measures of verbal fluency: FAS and animal naming. Arch Clin Neuropsychol. 1999 Feb;14(2):167-77. PMID: 14590600

^6^ Tombaugh TN. Trail Making Test A and B: normative data stratified by age and education. Archives of clinical neuropsychology : the official journal of the National Academy of Neuropsychologists. 2004;19(2):203-214

^7^ Heaton, R. K., Grant, I., & Matthews, C. G. (1991).Comprehensive norms for an expanded Halstead-ReitanBattery: Demographic corrections, research findings,and clinical applications. Odessa, FL: PsychologicalAssessment Resources.

**Supplementary Table 2.** Distribution of radiation doses to brain structures, stratified by field of radiotherapy. All values in units of Gy. Dxx = dose to xx% of the structure; Q1 = 1^st^ quartile; Q3 = 3^rd^ quartile; RT = radiotherapy; sd = standard deviation.

| Variable | Full Sample (n=30) | Focal only (n=19) | Whole brain ± focal boost (n=11) | p-value |
| --- | --- | --- | --- | --- |
| **Total RT Dose (RT prescription)** |  |  |  | 0.15 |
| Mean (sd) | 54.8 (6.5) | 56.2 (5.7) | 52.3 (7.2) |  |
| Median (Q1,Q3) | 54.0 (50.0,59.9) | 59.4 (50.0,60.0) | 54.0 (50.0,57.5) |  |
| Range (min, max) | (40,70.0) | (50,70) | (40,59.4) |  |
| **Brain mean** |  |  |  | **0.0033** |
| Mean (sd) | 24.3 (14.6) | 17.3 (6.7) | 36.3 (17.0) |  |
| Median (Q1,Q3) | 17.7 (13.1,38.0) | 16.5 (12.8,19.7) | 42.5 (36.0,46.9) |  |
| Range (min, max) | (2.8,50.4) | (8.0,31.2) | (2.8,50.4) |  |
| **Brain D50** |  |  |  | **0.0033** |
| Mean (sd) | 19.4 (16.5) | 11.0 (8.0) | 34.0 (17.4) |  |
| Median (Q1,Q3) | 13.8 (4.1,35.7) | 8.9 (4.0,14.4) | 39.9 (34.4,44.3) |  |
| Range (min, max) | (0.4,50.4) | (1.1,30.1) | (0.4,50.4) |  |
| **Infratentorial brain mean** |  |  |  | $<$**0.001** |
| Mean (sd) | 24.5 (18.3) | 14.9 (9.3) | 41.0 (18.5) |  |
| Median (Q1,Q3) | 19.7 (12.8,36.2) | 14.3 (7.5,21.0) | 49.6 (35.9,54.1) |  |
| Range (min, max) | (0.3,58.7) | (0.3,34.2) | (0.4,58.7) |  |
| **Left ventral frontal mean** |  |  |  | 0.12 |
| Mean (sd) | 28.9 (15.8) | 26.8 (15.6) | 32.6 (16.1) |  |
| Median (Q1,Q3) | 30.3 (21.8,39.1) | 25.4 (20.9,34.3) | 37.9 (33.2,40.9) |  |
| Range (min, max) | (0.5,59.8) | (0.5,59.8) | (0.5,50.4) |  |
| **Right ventral frontal mean** |  |  |  | 0.23 |
| Mean (sd) | 28.2 (16.1) | 26.1 (16.4) | 31.8 (15.7) |  |
| Median (Q1,Q3) | 32.7 (16.0,38.3) | 25.6 (15.3,36.6) | 37.8 (31.7,39.2) |  |
| Range (min, max) | (0.5,53.4) | (0.8,53.4) | (0.5,50.4) |  |
| **Left dorsofrontal mean** |  |  |  | **0.047** |
| Mean (sd) | 24.7 (17.6) | 18.9 (14.4) | 34.7 (18.6) |  |
| Median (Q1,Q3) | 22.8 (9.5,37.8) | 12.6 (8.7,27.1) | 37.7 (32.9,45.0) |  |
| Range (min, max) | (0.1,60.6) | (3.2,55.1) | (0.1,60.6) |  |
| **Right dorsofrontal mean** |  |  |  | **0.014** |
| Mean (sd) | 23.7 (16.4) | 18.1 (13.4) | 33.3 (17.1) |  |
| Median (Q1,Q3) | 22.5 (11.4,36.4) | 12.8 (9.5,24.6) | 38.0 (32.8,41.0) |  |
| Range (min, max) | (0.2,54.9) | (1.6,54.9) | (0.2,53.3) |  |
| **Bilat subcortical mean** |  |  |  | **0.047** |
| Mean (sd) | 30.0 (16.7) | 25.4 (15.0) | 38.0 (17.3) |  |
| Median (Q1,Q3) | 30.7 (16.9,43.1) | 22.6 (14.7,33.2) | 42.5 (37.5,48.6) |  |
| Range (min, max) | (0.4,55.0) | (2,55) | (0.4,54.0) |  |
| **Left parieto-occipital mean** |  |  |  | **0.002** |
| Mean (sd) | 20.5 (18.1) | 11.5 (11.0) | 36.2 (17.5) |  |
| Median (Q1,Q3) | 13.8 (3.7,40.5) | 10.4 (2.9,13.9) | 45.0 (35.1,46.4) |  |
| Range (min, max) | (1.2,50.4) | (2.0,43.8) | (1.2,50.4) |  |
| **Right parieto-occipital mean** |  |  |  | **0.0017** |
| Mean (sd) | 19.8 (17.3) | 10.7 (9.3) | 35.4 (17.1) |  |
| Median (Q1,Q3) | 14.2 (3.8,37.8) | 6.1 (3.4,14.9) | 42.0 (35.6,45.1) |  |
| Range (min, max) | (1.3,50.4) | (2.0,31.2) | (1.3,50.4) |  |
| **Left temporal mean** |  |  |  | **0.0033** |
| Mean (sd) | 26.5 (16.4) | 20.5 (12.5) | 37.0 (17.5) |  |
| Median (Q1,Q3) | 29.0 (14.2,38.4) | 21.2 (10.7,31.4) | 44.5 (36.3,47.7) |  |
| Range (min, max) | (0.7,52.5) | (0.7,37.6) | (2.4,52.5) |  |
| **Right temporal mean** |  |  |  | **0.0071** |
| Mean (sd) | 25.4 (16.1) | 19.7 (13.0) | 35.2 (16.5) |  |
| Median (Q1,Q3) | 23.3 (11.1,39.5) | 19.2 (8.5,27.3) | 40.0 (35.6,43.5) |  |
| Range (min, max) | (1.1,51.6) | (1.1,46.2) | (2.9,51.6) |  |
| **Left hippocampus mean** |  |  |  | **0.0082** |
| Mean (sd) | 29.9 (18.5) | 23.5 (15.0) | 40.9 (19.4) |  |
| Median (Q1,Q3) | 33.8 (14.9,45.6) | 20.1 (13.9,35.5) | 50.4 (38.4,53.6) |  |
| Range (min, max) | (0.7,58.3) | (0.7,50.5) | (2.2,58.3) |  |
| **Left hippocampus D50** |  |  |  | **0.031** |
| Mean (sd) | 29.0 (19.6) | 23.3 (17.5) | 39.7 (19.7) |  |
| Median (Q1,Q3) | 34.2 (10.5,46.5) | 21.0 (10.2,37.8) | 45.2 (38.3,54.0) |  |
| Range (min, max) | (0.5,57.2) | (0.5,53.5) | (2.4,57.2) |  |
| Missing | **1** | **0** | **1** |  |
| **Left hippocampus D40** |  |  |  | **0.045** |
| Mean (sd) | 30.6 (18.6) | 25.9 (16.9) | 39.5 (19.1) |  |
| Median (Q1,Q3) | 34.6 (15.2,48.0) | 21.5 (13.4,38.2) | 45.2 (38.4,52.8) |  |
| Range (min, max) | (0.7,57.3) | (0.7,56.9) | (2.5,57.3) |  |
| Missing | **1** | **0** | **1** |  |
| **Right hippocampus mean** |  |  |  | **0.0053** |
| Mean (sd) | 28.4 (17.3) | 22.0 (13.4) | 39.3 (18.3) |  |
| Median (Q1,Q3) | 27.5 (14.1,39.6) | 22.6 (12.0,29.4) | 47.7 (38.3,50.3) |  |
| Range (min, max) | (1.1,58.6) | (1.1,51.6) | (2.0,58.6) |  |
| **Right hippocampus D50** |  |  |  | **0.0061** |
| Mean (sd) | 27.6 (18.4) | 20.5 (14.5) | 39.7 (18.6) |  |
| Median (Q1,Q3) | 26.7 (12.0,42.0) | 20.2 (10.9,27.3) | 46.0 (38.6,51.1) |  |
| Range (min, max) | (1.0,58.5) | (1.0,54.3) | (1.9,58.5) |  |
| **Right hippocampus D40** |  |  |  | **0.019** |
| Mean (sd) | 30.6 (18.1) | 24.9 (15.5) | 40.5 (18.6) |  |
| Median (Q1,Q3) | 34.1 (14.2,43.7) | 25.9 (13.1,37.6) | 50.4 (38.7,51.9) |  |
| Range (min, max) | (1.1,58.7) | (1.1,56.3) | (2.1,58.7) |  |
| **Corpus callosum mean** |  |  |  | **0.014** |
| Mean (sd) | 28.1 (18.5) | 21.2 (17.9) | 39.9 (13.2) |  |
| Median (Q1,Q3) | 33.3 (9.6,44.1) | 13.4 (6.7,37.4) | 38.9 (36.9,47.8) |  |
| Range (min, max) | (3.7,55.7) | (3.7,54.5) | (7.0,55.7) |  |
| **Corpus callosum D50** |  |  |  | **0.026** |
| Mean (sd) | 28.4 (21.2) | 21.4 (22.0) | 40.3 (13.5) |  |
| Median (Q1,Q3) | 33.7 (8.6,43.2) | 9.5 (4.4,37.7) | 38.8 (36.9,45.2) |  |
| Range (min, max) | (1.4,60.6) | (1.4,60.6) | (9,60) |  |
| **Corpus callosum D40** |  |  |  | **0.03** |
| Mean (sd) | 29.4 (20.8) | 22.8 (21.7) | 40.9 (13.5) |  |
| Median (Q1,Q3) | 34.0 (9.9,44.8) | 13.6 (5.8,38.5) | 39.0 (36.9,46.8) |  |
| Range (min, max) | (1.8,60.9) | (1.8,60.9) | (9.6,60.4) |  |

**Supplementary Table 3**. Generalized estimating equation models of neurocognitive function across different cognitive domains, **after excluding one patient with baseline evaluation 42 weeks post-RT**. Negative coefficients (ß) denote worse performance associated with increasing magnitude of that variable. In this table for each cognitive domain, we included the structure that had the lowest q-value.

| **Cognitive domain** | **Evaluable** | **Baseline z-score** | | **Baseline function (z-score)** | | **Time since RT (years)** | | **Age at RT (years)** | | **Formal education (years)** | | **Dose to structure (per Gy)** | | | |
| --- | --- | --- | --- | --- | --- | --- | --- | --- | --- | --- | --- | --- | --- | --- | --- |
|  | n | Mean | SD | ß | p | ß | p | ß | p | ß | p | Structure | Metric | ß | q |
| Attention | 28 | -0.4 | 0.7 | None significant | | | | | | | | | | | |
| Executive function | 28 | -0.8 | 1.1 | -0.118 | 0.76 | -0.122 | 0.22 | -0.068 | **0.05** | 0.01 | 0.88 | Left parieto-occipital | Mean | -0.004 | 0.82 |
| Verbal abilities (language) | 29 | 0.2 | 0.9 | -0.313 | **<0.001** | 0.043 | **0.009** | -0.012 | **0.025** | 0.048 | **0.0019** | Left temporal | Mean | -0.008 | 0.31 |
| Motor dexterity | 24 | -1.2 | 1.6 | -0.348 | **<0.001** | -0.015 | 0.72 | -0.032 | **0.012** | 0.058 | 0.10 | Infratentorial brain | Mean | -0.011 | 0.28 |
| Speed | 28 | -0.5 | 1.3 | -0.121 | 0.13 | 0.031 | 0.27 | -0.018 | **0.013** | 0.046 | 0.11 | Left hippocampus | D40 | 0.009 | 0.53 |
| Verbal memory | 28 | -0.4 | 1.1 | -0.125 | 0.11 | -0.073 | **0.02** | -0.01 | 0.28 | 0.044 | **0.022** | Left dorsal frontal | Mean | 0.004 | 0.99 |
| Visual memory (model 1) | 28 | -0.6 | 1.0 | -0.494 | 0.051 | 0.033 | 0.48 | -0.017 | 0.11 | 0.003 | 0.94 | Subcortical | Mean | -0.011 | 0.96 |
| Visual memory (model 2) | 28 | -0.6 | 1.0 | -0.425 | 0.065 | 0.037 | 0.44 | -0.01 | 0.20 | 0.0005 | 0.99 | Left hippocampus | Mean | -0.005 | 0.96 |
| Visuospatial skills | 29 | 0.4 | 0.8 | None significant | | | | | | | | | | | |
| Working memory | 29 | 0.1 | 0.7 | -0.182 | 0.14 | -0.054 | **0.0059** | 0.002 | 0.66 | 0.049 | **<0.001** | Left ventral frontal | Mean | -0.004 | 0.83 |

**Supplementary Table 4**. Generalized estimating equation models of neurocognitive function across different cognitive domains, **considering only the 19 patients who received focal RT**. Negative coefficients (ß) denote worse performance associated with increasing magnitude of that variable. In this table for each cognitive domain, we included the structure that had the lowest q-value.

| **Cognitive domain** | **Evaluable** | **Baseline z-score** | | **Baseline function (z-score)** | | **Time since RT (years)** | | **Age at RT (years)** | | **Formal education (years)** | | **Dose to structure (per Gy)** | | | |
| --- | --- | --- | --- | --- | --- | --- | --- | --- | --- | --- | --- | --- | --- | --- | --- |
|  | n | Mean | SD | ß | p | ß | p | ß | p | ß | p | Structure | Metric | ß | q |
| Attention | 19 | -0.4 | 0.7 | None significant | | | | | | | | | | | |
| Executive function | 19 | -0.8 | 1.1 | -0.35 | **0.05** | -0.031 | 0.37 | -0.018 | 0.10 | 0.07 | **0.0053** | Left parieto-occipital | Mean | -0.018 | 0.11 |
| Verbal abilities (language) | 19 | 0.2 | 0.9 | -0.247 | **0.0024** | 0.072 | **0.0045** | 0.002 | 0.70 | 0.051 | **0.0028** | Left temporal | Mean | -0.012 | 0.43 |
| Motor dexterity | 15 | -1.2 | 1.6 | -0.326 | **0.0017** | -0.03 | 0.70 | -0.041 | **0.019** | 0.014 | 0.84 | Infratentorial brain | Mean | 0.018 | 0.68 |
| Speed | 19 | -0.5 | 1.3 | -0.172 | **0.0043** | 0.041 | 0.15 | -0.019 | **0.0036** | 0.063 | **0.002** | Left hippocampus | D40 | 0.014 | **0.017** |
| Verbal memory | 19 | -0.3 | 1.1 | -0.024 | 0.84 | -0.052 | 0.26 | -0.01 | 0.33 | 0.038 | 0.08 | Left dorsal frontal | Mean | -0.002 | 0.93 |
| Visual memory (model 1) | 19 | -0.6 | 0.9 | -0.098 | 0.52 | -0.005 | 0.92 | -0.004 | 0.64 | 0.025 | 0.37 | Subcortical | Mean | -0.011 | 0.59 |
| Visual memory (model 2) | 19 | -0.6 | 0.9 | -0.096 | 0.58 | -0.013 | 0.78 | 0.004 | 0.69 | 0.037 | 0.15 | Left hippocampus | Mean | -0.016 | 0.18 |
| Visuospatial skills | 19 | 0.4 | 0.8 | None significant | | | | | | | | | | | |
| Working memory | 19 | 0.1 | 0.7 | -0.221 | **0.011** | -0.013 | 0.67 | -0.001 | 0.90 | 0.064 | **<0.001** | Left ventral frontal | Mean | -0.014 | 0.20 |

**Supplementary Table 5**. Number of patients with a z-score decrease of given magnitude between first and last evaluation

|  | **z-score decrease** | | | |
| --- | --- | --- | --- | --- |
|  | ≥1.0 and <1.5 | ≥1.5 and <2.0 | ≥2.0 | Missing |
| **Attention** | 0 | 0 | 0 | 1 |
| **Executive function** | 1 | 0 | 1 | 1 |
| **Verbal abilities (language)** | 0 | 0 | 0 | 1 |
| **Motor dexterity** | 1 | 2 | 1 | 5 |
| **Speed** | 0 | 1 | 0 | 0 |
| **Verbal memory** | 2 | 0 | 0 | 1 |
| **Visual memory** | 0 | 0 | 1 | 1 |
| **Visuospatial skills** | 2 | 0 | 0 | 1 |
| **Working memory** | 2 | 0 | 0 | 0 |

**Supplementary Figure 2**. Plot of neurocognitive assessments for each study subject, relative to timing of RT.


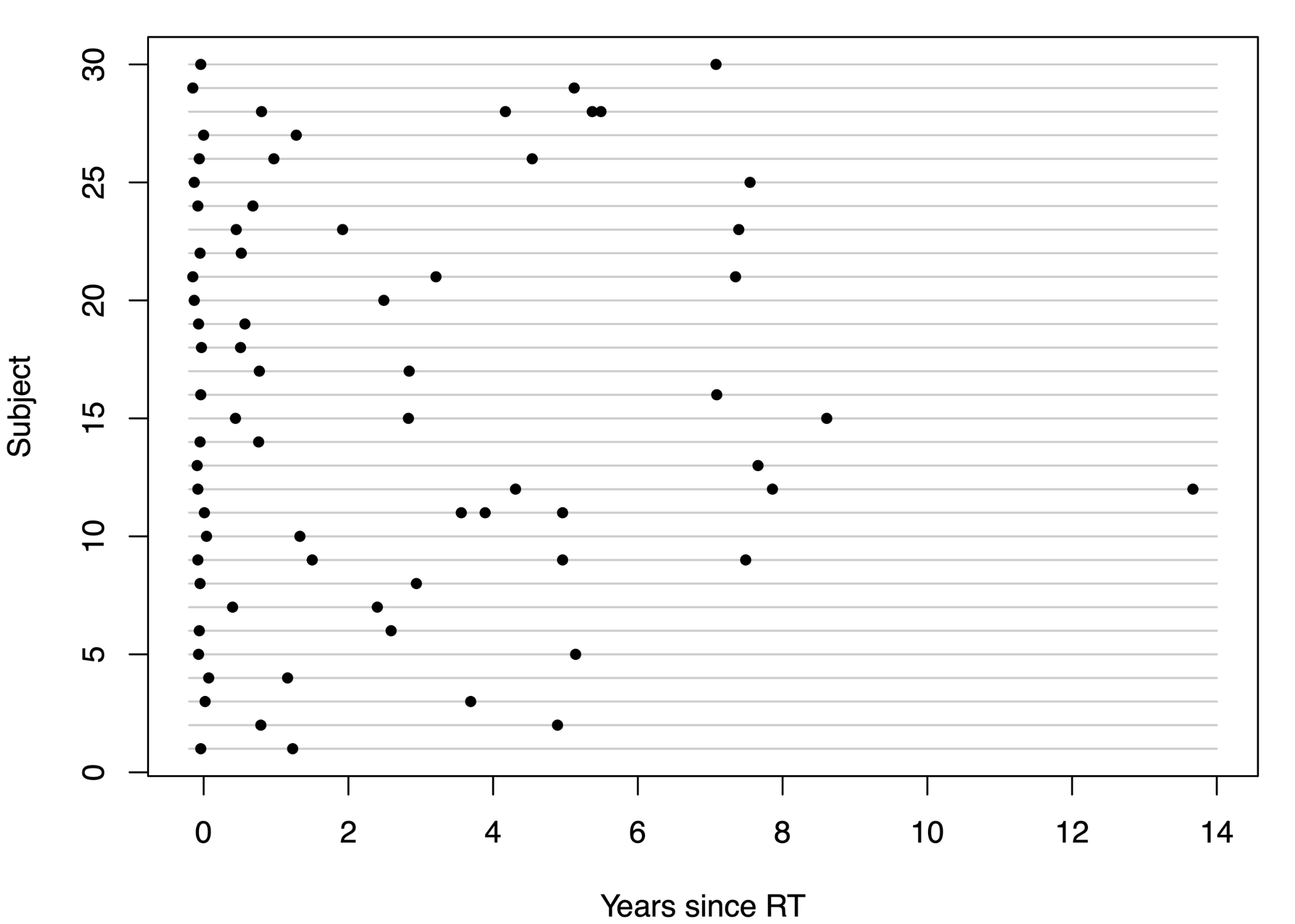


**Supplementary Figure 3**. Plots of neurocognitive domain z-scores over time since RT. Higher scores indicate better performance. A score of zero is equal to the population mean, with a population standard deviation of ±1 (gray shaded region).


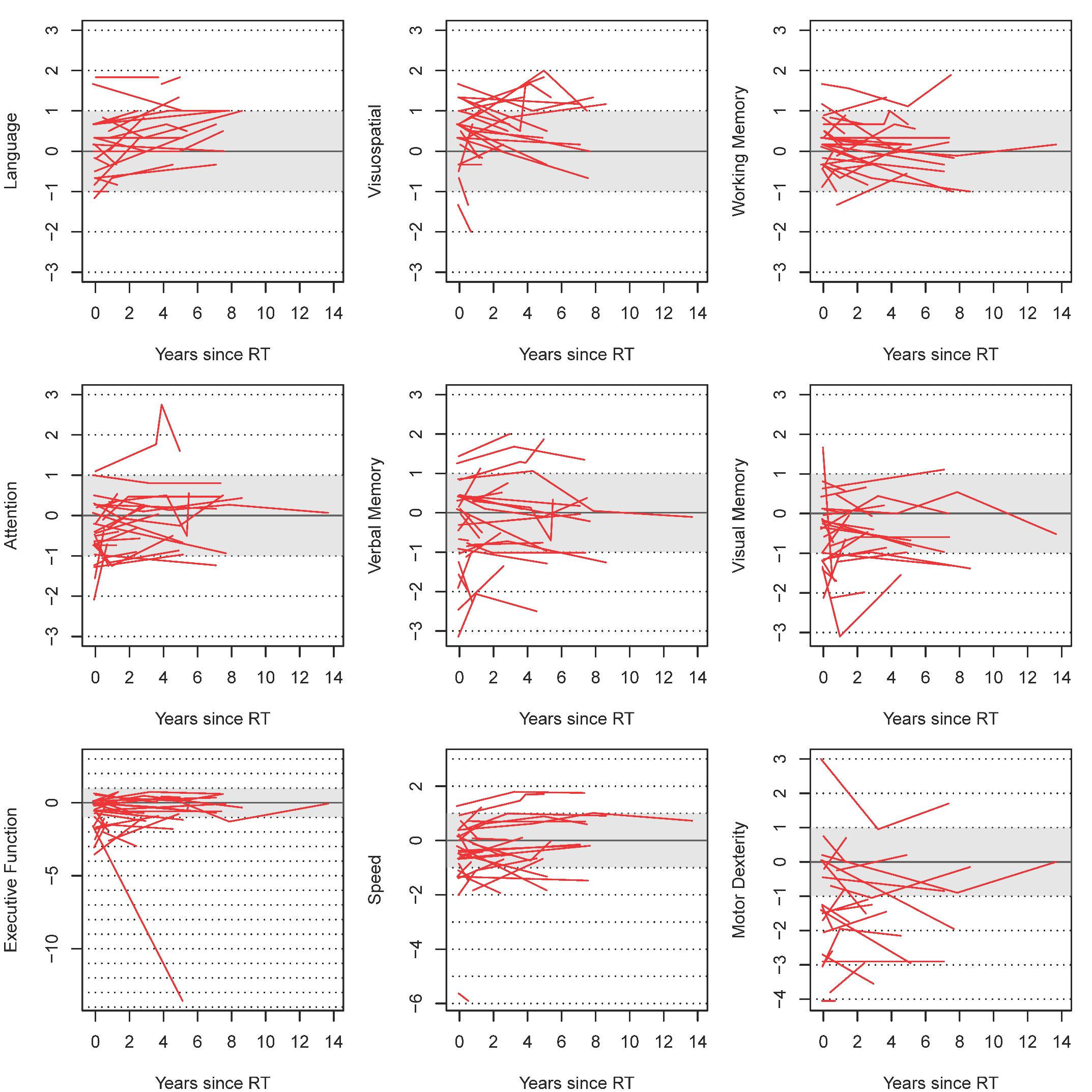

Supplement: Supplementary file 2 [file mmc2.docx]
